# Characteristics of intestinal bacteriophages and their relationship with Bacteria and serum metabolites during quail sexual maturity transition

**DOI:** 10.1186/s12917-024-03945-9

**Published:** 2024-03-08

**Authors:** Xinwei Xiong, Jishang Gong, Tian Lu, Liuying Yuan, Yuehang Lan, Xutang Tu

**Affiliations:** Jiangxi Provincial Key Laboratory of Poultry Genetic Improvement, Nanchang Normal University, Nanchang, Jiangxi 330032 China

**Keywords:** Quail, Gut microbiome, Bacteriophages, Metagenomic sequencing, Sexual maturity

## Abstract

**Background:**

Bacteriophages are prokaryotic viruses that rank among the most abundant microbes in the gut but remain among the least understood, especially in quails. In this study, we surveyed the gut bacteriophage communities in 22 quails at different ages (days 20 and 70) using shotgun metagenomic sequencing. We then systematically evaluated the relationships with gut bacteria and host serum metabolites.

**Results:**

We discovered that Myoviridae and Siphoviridae were the dominant bacteriophage families in quails. Through a random forest and LEfSe analysis, we identified 23 differential bacteriophages with overlapping presence. Of these, 21 bacteriophages (e.g., *Enterococcus phage IME-EFm5* and *Enterococcus phage IME-EFm1*) showed higher abundances in the day 20 group, while two bacteriophages (*Bacillus phage Silence* and *Bacillus virus WPh*) were enriched in the day 70 group. These key bacteriophages can serve as biomarkers for quail sexual maturity. Additionally, the differential bacteriophages significantly correlated with specific bacterial species and shifts in the functional capacities of the gut microbiome. For example, *Enterococcus* phages (e.g., *Enterococcus phage EFP01*, *Enterococcus phage IME-EFm5*, and *Enterococcus phage IME-EFm1*) were significantly (*P* < 0.001, FDR) and positively correlated with *Enterococcus faecalis*. However, the relationships between the host serum metabolites and either bacteriophages or bacterial species varied. None of the bacteriophages significantly (*P* > 0.05, FDR) correlated with nicotinamide riboside and triacetate lactone. In contrast, some differential bacterial species (e.g., *Christensenella massiliensis* and *Bacteroides neonati*) significantly (*P* < 0.05, FDR) correlated with nicotinamide riboside and triacetate lactone. Furthermore, characteristic successional alterations in gut bacteriophages, bacteria, and host serum metabolites across different ages highlighted a sexual maturity transition coexpression network.

**Conclusion:**

This study improves our understanding of the gut bacteriophage characteristics in quails and offers profound insights into the interactions among gut bacteriophages, bacteria, and host serum metabolites during the quail’s sexual maturity transition.

**Supplementary Information:**

The online version contains supplementary material available at 10.1186/s12917-024-03945-9.

## Background

Emerging data suggest that viruses are integral members of the host microbiome, populating surfaces such as the gut, mouth, and skin [1–3]. The majority of these viruses that target bacteria are bacteriophages, often exhibiting species-level specificity [4]. Bacteriophages harbor numerous genetic functions, including platelet binding, complement and immunoglobulin degradation, and other gene functions, potentially offering significant advantages to their bacterial hosts [2, 5]. Moreover, the metagenomic composition of bacteriophages has been linked with diseases, such as malnutrition and inflammatory bowel disease [6, 7]. However, the actual behavior of bacteriophages in the gut, particularly in quails, remains undiscovered.

Bacteriophages are the most prolific biological entities on earth and significantly influence microbial communities [8, 9]. Their communities also react to disturbances, albeit differently than one might anticipate based on the responses of their bacterial hosts [10]. Bacteriophages can destroy host cells, alter host phenotypes via lysogenic conversion, and change bacterial communities through infection [11]. By influencing the stability of the intestinal microbiota, bacteriophages can mold the immunological and metabolic functions of the intestine [12]. Additionally, the bacteria-phage interactions and host-microbial relationships during the weaning transition affect host metabolism, resulting in advantageous host adaptations across the three weaning phases [4]. These studies indicate that phages play a pivotal role in shaping the gut bacterial community structure, and bacteria-phage interactions are crucial to bacterial physiology and metabolism.

Quails are significant poultry breeds and serve as essential sources of eggs and meat for human consumption. To date, few studies have concentrated on the alterations in the gut microbiome during quail sexual maturity. Our previous research identified five species that were abundant in the day 20 group (e.g., *Enterococcus faecalis*) and 12 species that were prevalent in the day 70 group (e.g., *Bacteroides neonati* and *Christensenella massiliensis*) as key bacterial markers for quail sexual maturity [13]. However, the quail bacteriophage interactions, bacteria-phage dynamics, and bacteriophage-serum metabolites interactions during sexual maturity transition remains unreported. In the present study, we gathered 22 fecal samples from quails at days 20 and 70 during sexual maturity transition phases and performed shotgun metagenomic sequencing to exhaustively detail gut bacteriophage composition throughout this transition. Moreover, we also devised a co-occurrence network based on the relative abundances of different gut bacteriophages, bacterial species, and host serum metabolites during sexual maturity transition, aiming to further elucidate the intricacies of bacteria-phage interactions and their influence on host metabolism.

## Results

### Taxonomic characterization of quail Gut bacteriophage during sexual maturity transition

**Fig. 1:**
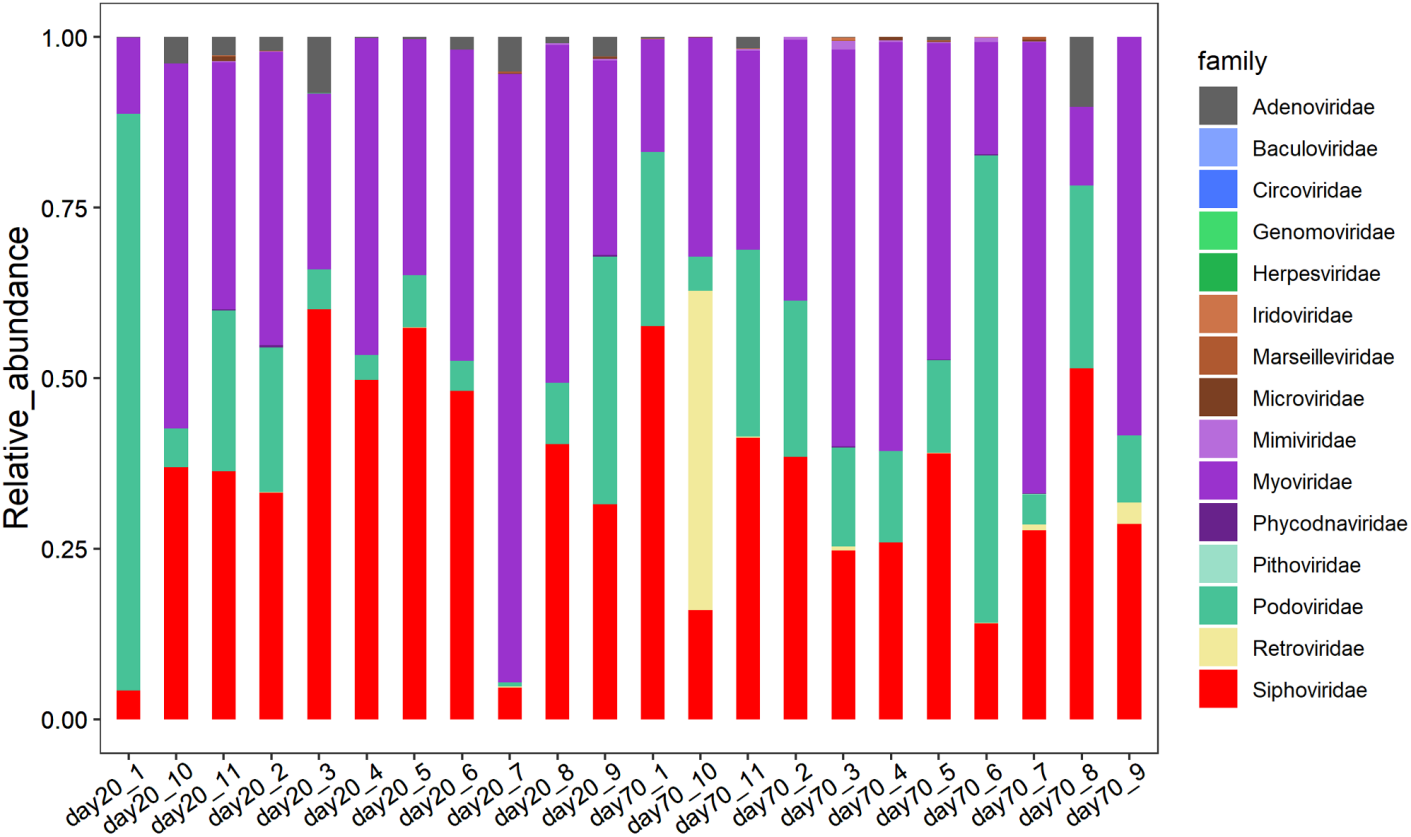
Categories and relative abundance of gut bacteriophages at the family level based on shotgun metagenomic sequencing data from all tested samples

**Fig. 2:**
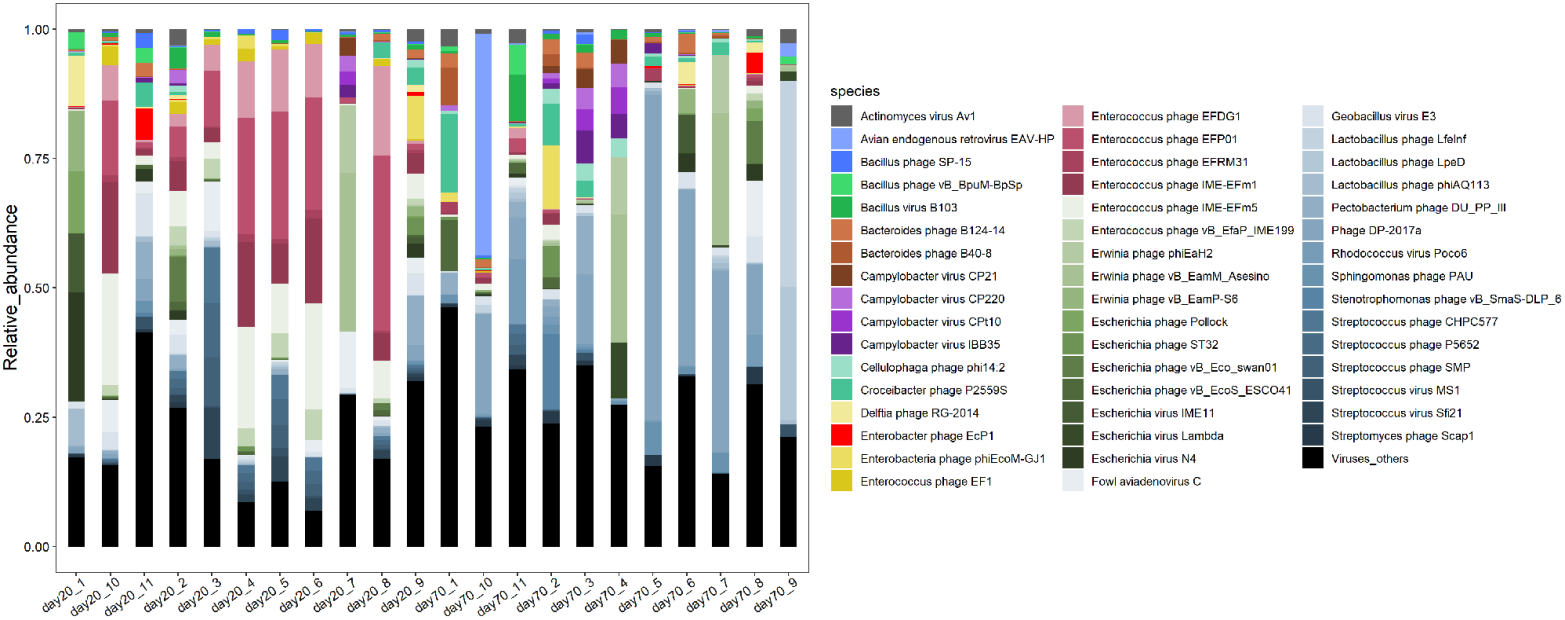
Categories and relative abundance of gut bacteriophages at the species level based on shotgun metagenomic sequencing data from all tested samples

To probe the gut bacteriophage compositions of quails, we performed shotgun metagenomic sequencing on 22 fecal samples. The phylogenetic composition of the fecal bacteria was ascertained by performing a BLAST search against the National Center for Biotechnology Information (NCBI) non-redundant (NR) database. Within the 22 samples, a collective 15 families and 94 genera were identified. Myoviridae (spanning 11.15–89.26%) and Siphoviridae (spanning 4.19–60.10%) emerged as the two most predominant bacteriophage families in the quail gut microbiome (Fig. 1). At the genus level, the most prevalent genera in quails were *Cp220virus* and *Aviadenovirus*, boasting average abundances across samples of 11.73% and 9.33%, respectively, followed by *N4virus* and *Sfi21dt1virus*. At the species level, a sum of 515 bacteriophage species were detected in the 22 samples. Among these, 22 species were discerned in over 90% of the analyzed samples, thus being categorized as core bacteriophages. *Phage DP-2017a* was the most abundant bacteriophage in the inspected samples, followed by *Enterococcus phage EFP01* and *Enterococcus phage IME-EFm5* (Fig. 2).

### Bacteriophage differences during quail sexual maturity transition

**Fig. 3:**
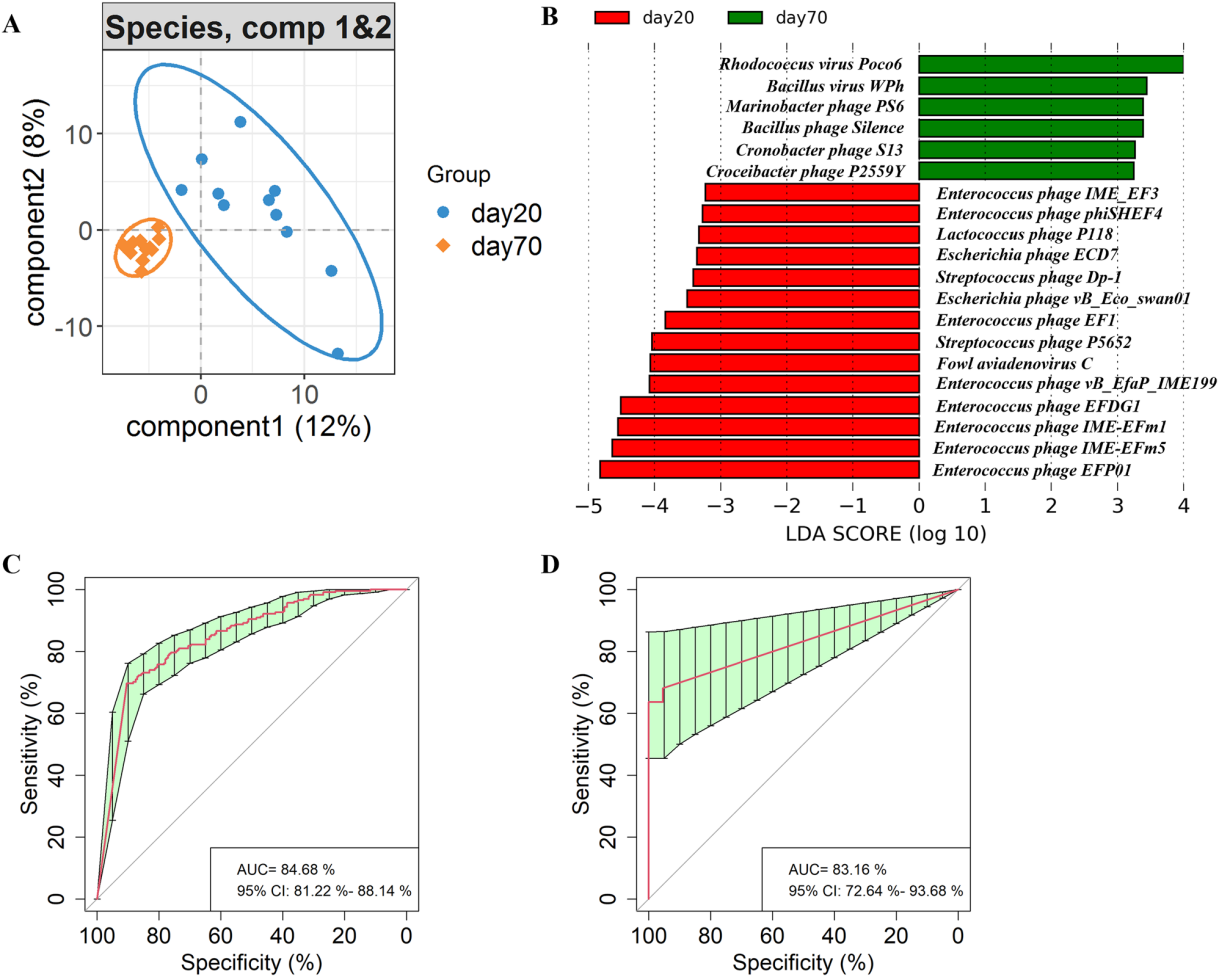
Characteristics of gut bacteriophages between the day 70 and day 20 groups. (**A**) PLS-DA plot highlighting significantly different gut bacteriophage species between the day 70 and day 20 groups. (**B**) Differential gut bacteriophage species between the day 70 and day 20 groups. (**C**) and (**D**) Receiver operating curves (ROC) for the day 20 and day 70 groups, respectively

To discern bacteriophage species associated with sexual maturity, we examined the differences in bacteriophage composition of the quail gut microbiome between the day 70 and day 20 cohorts. The Partial Least Squares-Discriminant analysis (PLS-DA) depicted a discernible shift in gut bacteriophages between day 70 and day 20 groups (Fig. 3A). As anticipated, linear discriminant analysis (LDA) effect size (LEfSe) analysis pinpointed 55 differential bacteriophages (Fig. 3B and Supplementary Table S1), including 49 species manifesting elevated abundance in the day 20 group and 6 species prominently enriched in the day 70 group. The day 20 group predominantly featured bacteriophages from the Siphoviridae family (21 species, e.g., *Enterococcus phage IME-EFm1*, *Enterococcus phage IME-EFm5*, and *Lactobacillus phage Lrm1*), Podoviridae (9 species, e.g., *Enterobacteria phage 285P*, *Enterobacteria phage EcoDS1*, and *Escherichia phage vB_EcoP_F*), and Myoviridae (9 species, e.g., *Bacillus virus Bcp1*, *Cronobacter virus PBES02*, and *Staphylococcus virus G1*). Conversely, the day 70 group saw enrichment of species such as *Rhodococcus virus Poco6*, *Bacillus virus WPh*, *Croceibacter phage P2559Y*, *Bacillus phage Silence*, *Cronobacter phage S13*, and *Marinobacter phage PS6*. Moreover, a random forest analysis was deployed to pinpoint bacteriophage biomarkers that differentiated quail sexual maturity at the species level (Supplementary Fig. 1 and Supplementary Table S2). This unveiled that 28 bacteriophage species were significantly distinct between the day 70 and day 20 cohorts, of which, 23 coincided with the LEfSe analysis (Supplementary Table S3). These overlapping bacteriophages enriched in either the day 20 or day 70 group served as potent markers differentiating the sexual maturity of quails boasting diagnostic accuracies of 84.68% and 83.16% respectively, as evidenced by the area under the curve (AUC) (Fig. 3C and D).

### Bacteriophages associated with bacterial species during quail sexual maturity transition

**Fig. 4:**
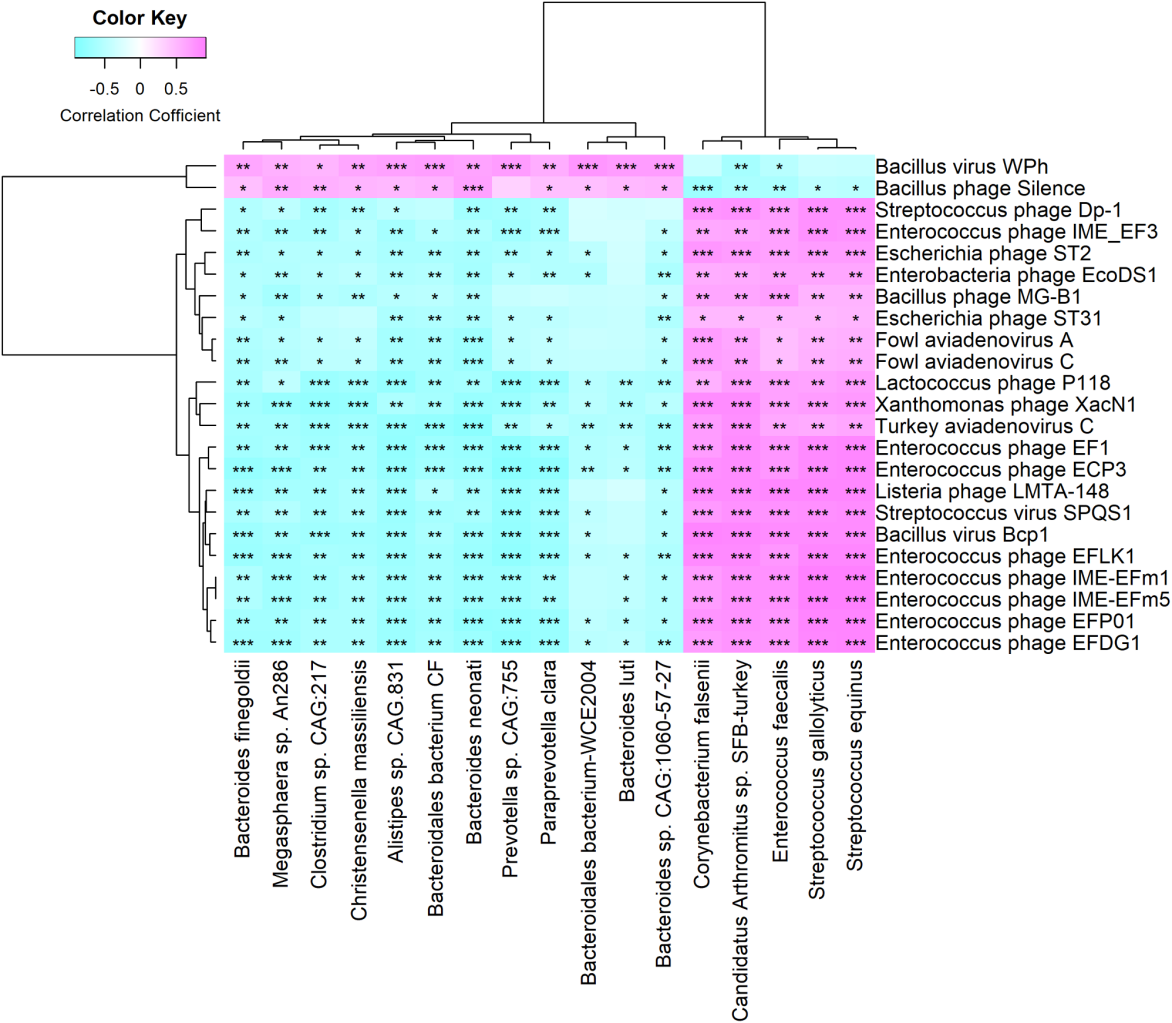
Heat maps depicting relationships between differential gut bacteriophage species and differential bacterial species. The X-axis represents bacterial species, while the Y-axis indicates bacteriophage species. Significance levels are indicated as follows: *P < 0.05, **P < 0.01, and ***P < 0.001

In our previous study, 17 bacterial species, 21 KEGG pathways, and 25 CAZymes exhibited significant differences between the day 20 and day 70 groups. Specifically, five species were enriched on day 20 (e.g., *Enterococcus faecalis*) while12 species were prevalent on day 70 (e.g., *Bacteroides neonati*, *Clostridium sp. CAG:217*, and *Christensenella massiliensis*). These bacterial species showed significant correlations with specific KEGG pathways and CAZymes. To thoroughly evaluate the relationship between the bacteriophages and these differential bacterial species, we employed Spearman correlation analysis. Our findings showed that the bacterial species predominant in the day 20 group had significant (*P* < 0.05, FDR) and positive correlations with the bacteriophages predominant in the same day 20 group (Fig. 4). For instance, *Enterococcus faecalis* showed a significant (*P* < 0.001, FDR) and positive correlation with *Enterococcus* phages (e.g., *Enterococcus phage EFP01*, *Enterococcus phage IME-EFm5*, and *Enterococcus phage IME-EFm1*). *Streptococcus gallolyticus* and *Streptococcus equinus* significantly (*P* < 0.001, FDR) and positively correlated with *Streptococcus phage Dp-1* and *Streptococcus virus SPQS1*. For the day 70 group, *Bacillus virus WPh* had a significant (*P* < 0.05, FDR) and positive correlation with the bacterial species prevalent on day 70, and *Bacillus phage Silence* was significantly (*P* < 0.05, FDR) and positively correlated with most of the bacterial species (11 out of 12, excluding *Prevotella sp. CAG:755*) enriched on day 70 (Fig. 4). Additionally, the majority (15 out of 23) of the bacteriophages (e.g., *Enterococcus phage EFLK1* and *Enterococcus phage ECP3*) had a significant (*P* < 0.05, FDR) correlation with all KEGG pathways (Supplementary Fig. 2). Pertaining to CAZymes, we found that only *Enterococcus phage IME-EFm1*, *Enterococcus phage IME-EFm5*, *Turkey aviadenovirus C*, *Xanthomonas phage XacN1*, and *Escherichia phage ST2* significantly (*P* < 0.05, FDR) correlated with all CAZymes (Supplementary Fig. 3).

### Host serum metabolites associated with bacteriophages during quail sexual maturity transition

**Fig. 5:**
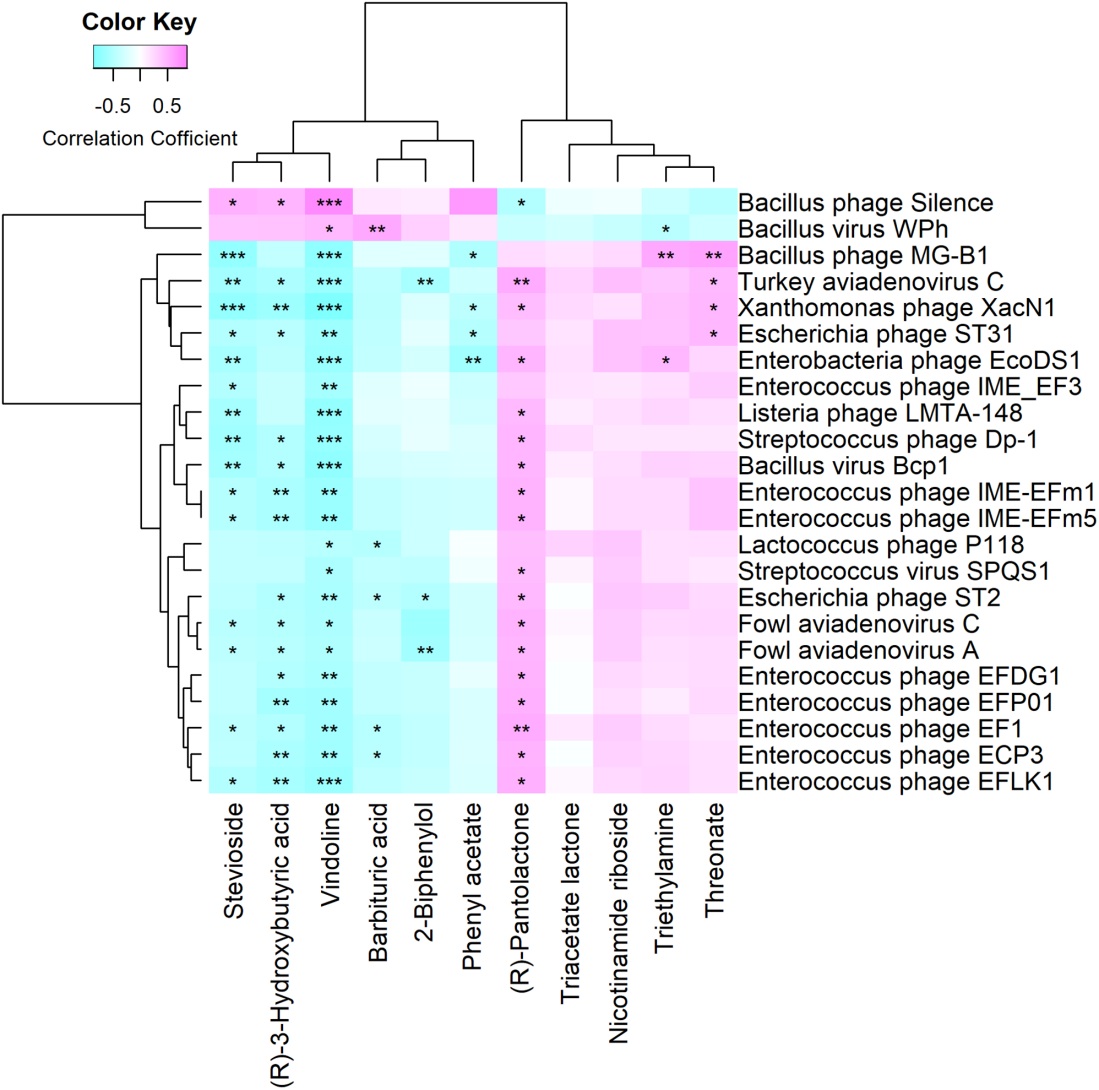
Heat maps illustrating the relationships between differential gut bacteriophage species and differential serum metabolites. The X-axis denotes serum metabolites, while the Y-axis refers to bacteriophage species. Significance levels are denoted as: *P < 0.05, **P < 0.01, and ***P < 0.001

In our previous study, we identified a total of 11 metabolites that displayed distinct enrichment patterns between the day 20 and day 70 groups. Specifically, nicotinamide riboside, (R)-pantolactone, triacetate lactone, triethylamine, and threonate were more abundant in the day 20 group, while D-ribose, stevioside, vindoline, phenyl acetate, (R)-3-hydroxybutyric acid, and barbituric acid were more prominent in the day 70 group. In this study, we explored the correlation between the shifts in bacteriophages and the alterations in serum metabolites using Spearman correlation analysis. Our findings revealed that vindoline significantly (*P* < 0.05, FDR) correlated with all the identified bacteriophages (Fig. 5). Moreover, most of (17/21) the bacteriophages enriched in the day 20 group exhibited a significant (*P* < 0.05, FDR) positive correlation with (R)-pantolactone, which was also predominant in the day 20 group. In particular, *Enterococcus phage IME_EF3* which was abundant in the day 20 group showed a significant (*P* < 0.05, FDR) negative correlation with vindoline and stevioside, both of which were enriched in the day 20 group. This mirrored the patterns observed with the bacterial species *Enterococcus faecalis*. Nonetheless, none of the bacteriophages exhibited a significant (*P* < 0.05, FDR) correlation with nicotinamide riboside and triacetate lactone (Fig. 5). As for differential bacterial species, triacetate lactone had a significant (*P* < 0.05, FDR) correlation with *Christensenella massiliensis* and *Clostridium sp. CAG:217*. Similarly, nicotinamide riboside showed significant (*P* < 0.05, FDR) correlations with multiple bacterial species, including *Bacteroides neonati*, *Bacteroides luti*, *Bacteroides finegoldii*, *Bacteroidales bacterium WCE2004*, *Bacteroidales bacterium CF*, *Bacteroides sp. CAG:1060_57_27*, *Alistipes sp. CAG:831*, and *Megasphaera sp. An286*.

### Co-occurrence analysis among the gut bacteriophages, bacteria, and serum metabolites

**Fig. 6:**
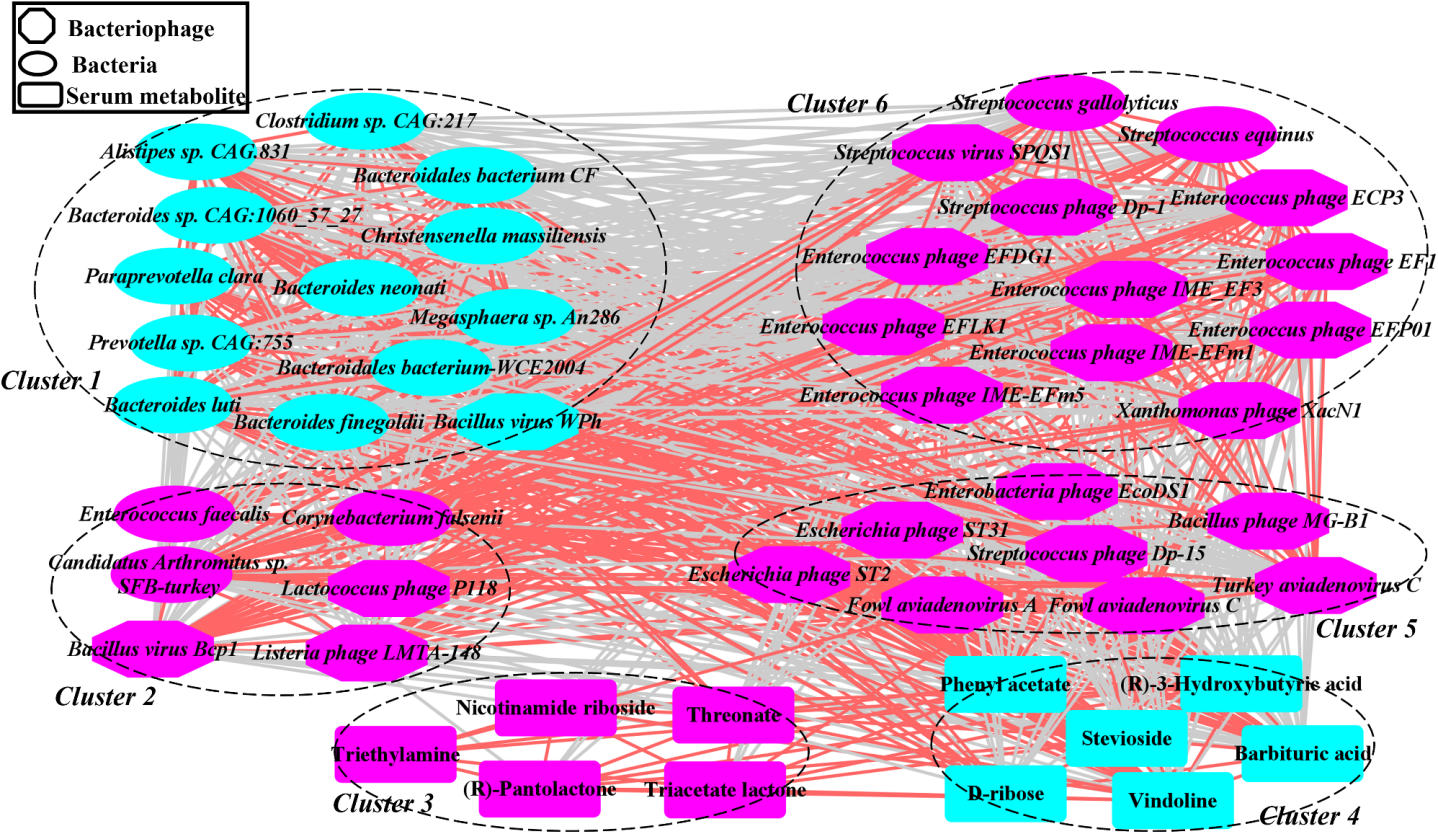
A co-occurrence network built from gut bacteriophages, bacterial species, and host serum metabolites, highlighting differences in abundance between the day 70 and day 20 groups. The purple and blue dots represent the day 20 and day 70 groups, respectively. Edges between nodes depict Spearman’s negative (light gray) or positive (light red) correlations

We delved further into the potential correlations among gut bacteriophages, host serum metabolites, and differential bacterial species by using co-occurrence network analysis. The analysis revealed that the gut bacteriophages, host serum metabolites, and differential bacterial species predominantly formed six clusters, exhibiting robust and extensive co-occurrence relationships (Fig. 6). Gut bacteriophages predominant in the day 20 group were chiefly situated in clusters 5 and 6. In contrast, differential bacterial species more common in the day 70 group were predominantly found in cluster 1. Additionally, serum metabolites abundant in the day 20 group populated cluster 3, while cluster 4 comprised six host serum metabolites that were more prevalent in the day 70 group. Notably, *Bacillus virus WPh*, which was enriched in the day 70 group, displayed a positive and significant correlation with bacterial species (e.g., *Christensenella massiliensis*, *Clostridium sp. CAG:217*, and *Bacteroides neonati*) located in cluster 1. In contrast, *Streptococcus gallolyticus* and *Streptococcus equinus*, which were more abundant in the day 20 group, showed a positive and significant correlation with bacteriophages (e.g., *Streptococcus phage Dp-1* and *Streptococcus virus SPQS1*) found in cluster 6.

## Discussion

Bacteriophages play multifaceted roles in microbial evolution and ecology [14]. Although the bacterial components of the microbiome have garnered considerable attention, the composition and physiological significance of bacteriophages, especially in quails remain relatively unexplored. In this study, we systematically assessed the impact of host sexual maturity on the gut bacteriophage composition in quails aged between 20 and 70 days using metagenomic sequencing data. We also examined the relationships between bacteriophages, different bacterial species, functional capacities of the gut microbiome, and host serum metabolites. To the best of our knowledge, this study is the first to delve into the interplay among bacteriophages, bacterial species, and host serum metabolites.

In the current study, Myoviridae and Siphoviridae emerged as the two predominant bacteriophage families in quails. Phages within the Myoviridae and Siphoviridae families share an evolutionary lineage with other cellular biological entities that possess the shared function of penetrating bacterial envelopes [15]. Furthermore, Myoviridae employ double-layered contractile tails to infect bacteria [16] and are primarily shaped by vertical evolution as opposed to horizontal gene transfer [17, 18]. Our analysis, using LEfSe and random forest, identified 23 differential bacteriophages, of which eight were *Enterococcus* phages. Combinations of *Enterococcus* phages have demonstrated greater efficacy than single phages in thwarting the proliferation of antibiotic- and phage-resistant *Enterococcus* mutants in vitro [19, 20]. Notably, we observed that *Enterococcus* phages significantly (*P* < 0.001, FDR) and positively correlated with *Enterococcus faecalis*. Additionally, *Streptococcus gallolyticus* and *Streptococcus equinus* displayed significant positive correlations with *Streptococcus phage Dp-1* and *Streptococcus virus SPQS1*, respectively (*P* < 0.001, FDR). A previous study by Patterson and Burkholder revealed that several microbial species (e.g., *Enterococcus*, and *Streptococcus*) used as probiotics can diminish poultry mortality across various ages, and enhance growth rates and feed efficiency [21]. These findings suggest that bacteria-phage interactions could be pivotal for quail adaptation during the sexual maturity transition.

Spearman correlation analysis revealed that all differential bacteriophages significantly correlated with vindoline (*P* < 0.05, FDR). Vindoline, a monomeric Vinca alkaloid, is a natural monomer known for its anti-inflammatory properties [22, 23]. Specifically, the *Enterococcus phage IME_EF3* shared a correlation with *Enterococcus faecalis* in the day 20 cohort. This phage possesses an isometric head and a lengthy non-contractile tail, with a 41 kb linear double-stranded DNA genome encoding 69 putative proteins, 32 of which have annotated function [24]. However, *Enterococcus phage IME_EF3* is not apt for phage therapy against *Enterococcus faecalis* unless one could excise the metallo-beta-lactamase gene; however, its lysin might prove beneficial in treating *Enterococcus faecalis* infections [24]. Contrarily, unlike the differential bacterial species, none of the bacteriophages displayed a significant correlation with nicotinamide riboside or triacetate lactone (*P* > 0.05, FDR). Nicotinamide ribose is known to ameliorate metabolic dysfunctions and lower various inflammatory cytokine levels [25]. These observations imply that bacteriophage-host and bacteria-host interactions may differ, but both are crucial for quail adaptation during their sexual maturity transition.

## Conclusion

In summary, we observed significant alterations in gut bacteriophage composition during the sexual maturity processes in quails. A total of 23 differential bacteriophages were identified using LEfSe and random forest analysis, serving as the key biomarkers for quail sexual maturity. These differential bacteriophages notably correlate with specific bacterial species. The differential bacteriophages and bacterial species share correlations with KEGG pathways and CAZymes, but not with serum metabolites. This study provides valuable insights into the interactions among gut bacteriophages, bacteria, and serum metabolites in quails. Nevertheless, the causality and underlying mechanisms remain undefined, which will be the focus of future research.

## Methods

### Sample collection and microbial DNA extraction

In this study, we incorporated a total of 22 quail samples from Jiangxi Hengyan Poultry Co. Ltd. (Fengcheng, Jiangxi, China), including 10 Japanese quails (7 females and 3 males) and 12 Hengyan white feather quails (6 females and 6 males). The quails were given *ad libitum* access to clean water and a commercial formula (Supplementary Table S4), and were maintained under uniform management conditions and environment. Based on previous research [26], we designated quails aged 20 days old as representing the pre-sexual maturity stage and those aged 70 days old as the post-sexual maturity stage, including 11 quails aged 20 days and 11 quails aged 70 days. Fresh fecal samples were obtained using a rectal kneading method from each quail on days 20 and 70. Throughout our study, all quails remained in a healthy condition, with no evident illnesses or medical treatments from birth to the conclusion of our research. The gathered fecal samples were promptly frozen in liquid nitrogen for transit, and subsequently stored at − 80 °C until further analysis. Blood samples were simultaneously collected from each quail.

Microbial DNA from the 22 quail samples was extracted utilizing the QIAamp Fast DNA Stool Mini Kit (Qiagen, Germany), according to the manufacturer’s instructions. The DNA concentration and quality was assessed by a Nanodrop-2000 spectrophotometer (Thermo Fisher Scientific, MA, US) and 0.8% (w/v) agarose gel electrophoresis, respectively. All extracted fecal microbial DNA samples were preserved at -20 °C for future use.

### Metagenomic sequencing and analysis

Metagenomic sequencing of all 22 microbial DNA samples was carried out on the Novaseq-PE150 platform. Following the manufacturer’s instructions, we generated a paired-end sequencing library with an insert size of 350 bp using the NEB Next® Ultra™ DNA Library Prep Kit (NEB, USA). Index codes were appended to attribute the sequences to their corresponding samples. The Agilent 2100 Bioanalyzer facilitated the evaluation of the library’s size distribution. Subsequently, the libraries were sequenced and quantified on Novaseq 6000 platform (Illumina, USA) and real-time PCR.

To curate the raw sequencing data, we eliminated adapters and purged low-quality reads. The Bowtie2.2.4 software [27] was then employed to remove host contamination by referencing against the quail genome (INSDC: LSZS01000000). Clean data assembly was achieved using the SOAPdenovo software (v.2.21) [28, 29]. MetaGeneMark (V2.10) [29, 30] was used to predict open reading frames (ORF) with the contigs longer than 500 bp. The gene catalog (Unigenes) was derived by eliminating redundant genes from all predicted ORFs via the Cluster Database at High Identity with Tolerance (CD-HIT) software [31]. These Unigenes were then aligned to bacteriophage sequences extracted from the NCBI NR database using the DIAMOND software [32]. Results were refined to include only those with an e value ≤  the smallest e value ×10 [33], using the LCA algorithm incorporated into the MEGAN [34] classification software.

### Metabolomic profile determination of quail serum samples

As described in our previous study [13], the untargeted metabolomics was used to determine the metabolomic profiles of all 22 quail serum samples. A mixture of 400 µL precooled methanol and 100 µL quail serum was vortexed. LC-MS/MS analysis was then performed using a Vanquish UHPLC system (Thermo Fisher) coupled to an Orbitrap Q Exactive series mass spectrometer (Thermo Fisher Scientific). Raw data files generated by UHPLC-MS/MS were processed using Compound Finder 3.0 (CD3.0, Thermo Fisher), to determined lignment, peak pickup, and quantification of each metabolite. R (R version R-3.4.3), Python (Python 2.7.6 version), and CentOS (CentOS release 6.6) software was used to perform subsequent statistical analysis.

### Statistical analysis

Linear discriminant analysis (LDA) and effect size (LEfSe) analysis were performed under the criterion of α = 0.01, and an LDA score threshold of at least 2.50 [35]. Random forest analysis incorporating adjusted settings (ntree = 1000) was employed to pinpoint gut bacteriophage species pivotal for distinguishing sexual maturity stages in quails [36]. The associations between gut bacteriophage species and differential bacterial species, differential KEGG pathways, differential CAZymes, and host serum metabolites were gauged via Spearman correlation analysis. Multiple tests were adjusted using Story’s false discovery rate (FDR). Partial least squares-discriminant analysis (PLS-DA) was undertaken to contrast gut bacteriophage species between the day 70 and day 20 cohorts [37]. Co-abundance (indicated as positive) and co-exclusion (represented as negative) relationships among differential gut bacteriophages, bacterial species, and host serum metabolites were discerned through Sparse correlations for compositional data (SparCC) [35], predicated on their relative abundance metrics. For a visual representation of the intricate relationships, network analysis was orchestrated and visualized in Cytoscape (v 3.6.1) [38].

### Electronic supplementary material

Below is the link to the electronic supplementary material.

**Supplementary Material 1: Figure S1.** Random Forest analysis to determine our ability to discriminate samples from different sexual maturity periods based on gut bacteriophage species

## Data Availability

The metagenomic sequence datasets provided in this study can be found in the online repository. The name and login number of the repository are as follows: NCBI SRA Bioproject, login number: PRJNA861719.

## Abbreviations

AUC: area under the curve
bp: base-pairs
CAZy: Carbohydrate-Active enZYmes database
CD-HIT: Cluster Database at High Identity with Tolerance
FDR: false discovery rate
NCBI: National Center for Biotechnology Information
NR: non-redundant
ORF: open reading frames
PLS-DA: Partial least squares-discriminant analysis
KEGG: Kyoto Encyclopedia of Genes and Genomes
SparCC: Sparse Correlations for Compositional
LDA: linear discriminant analysis
LEfSe: linear discriminant analysis effect size
