# Supplementary material for: Characteristics of intestinal bacteriophages and their relationship with Bacteria and serum metabolites during quail sexual maturity transition
Source: BMC Vet Res. 2024 Mar 8;20:93. doi: 10.1186/s12917-024-03945-9 (PMC10921806; doi:10.1186/s12917-024-03945-9)
Supplement: Supplementary file 1 — Supplementary Material 1: Figure S1. Random Forest analysis to determine our ability to discriminate samples from different sexual maturity periods based on gut bacteriophage species [file 12917_2024_3945_MOESM1_ESM.docx]

**Supporting information**

**Characteristics of Intestinal Bacteriophages and their Relationship with Bacteria and Serum Metabolites during Quail Sexual Maturity Transition**

*Xinwei Xiong^†^*, Jishang Gong^†^, Tian Lu, Liuying Yuan, Yuehang Lan, and Xutang Tu**

*Jiangxi Provincial Key Laboratory of Poultry Genetic Improvement,* *Nanchang Normal University, Nanchang, Jiangxi 330032, China.*

† These authors contributed equally to this work.

*Corresponding author: Xinwei Xiong and Xutang Tu

Jiangxi Provincial Key Laboratory of Poultry Genetic Improvement, Nanchang Normal University, Nanchang 330032, China.

Phone: 0086-791- 83812115

Fax: 0086-791- 83812115

E-mail: XinweiXiong@hotmail.com and tuxutang@ncnu.edu.cn


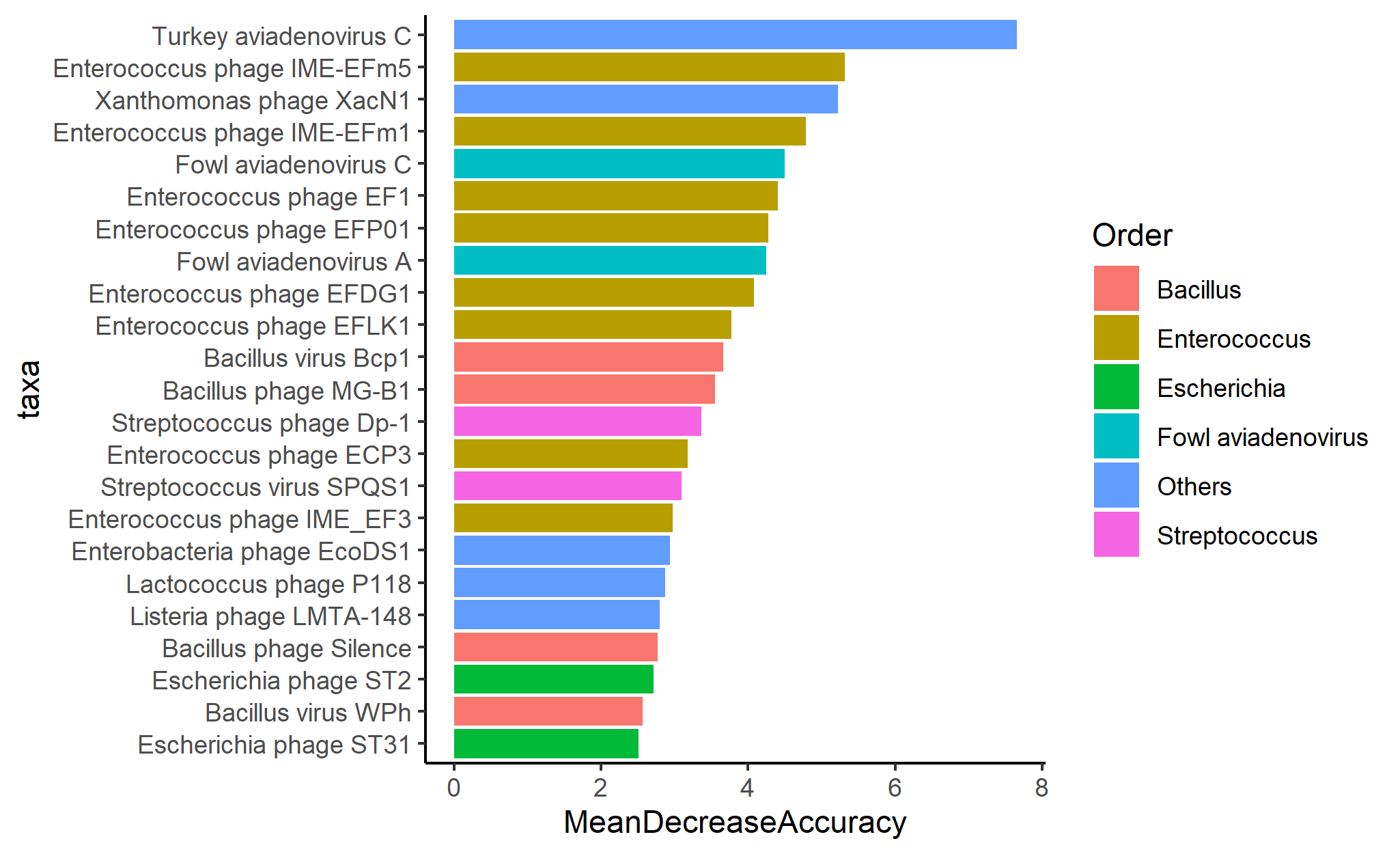


**Supplementary Figure 1** Random Forest analysis to determine our ability to discriminate samples from different sexual maturity periods based on gut bacteriophage species.


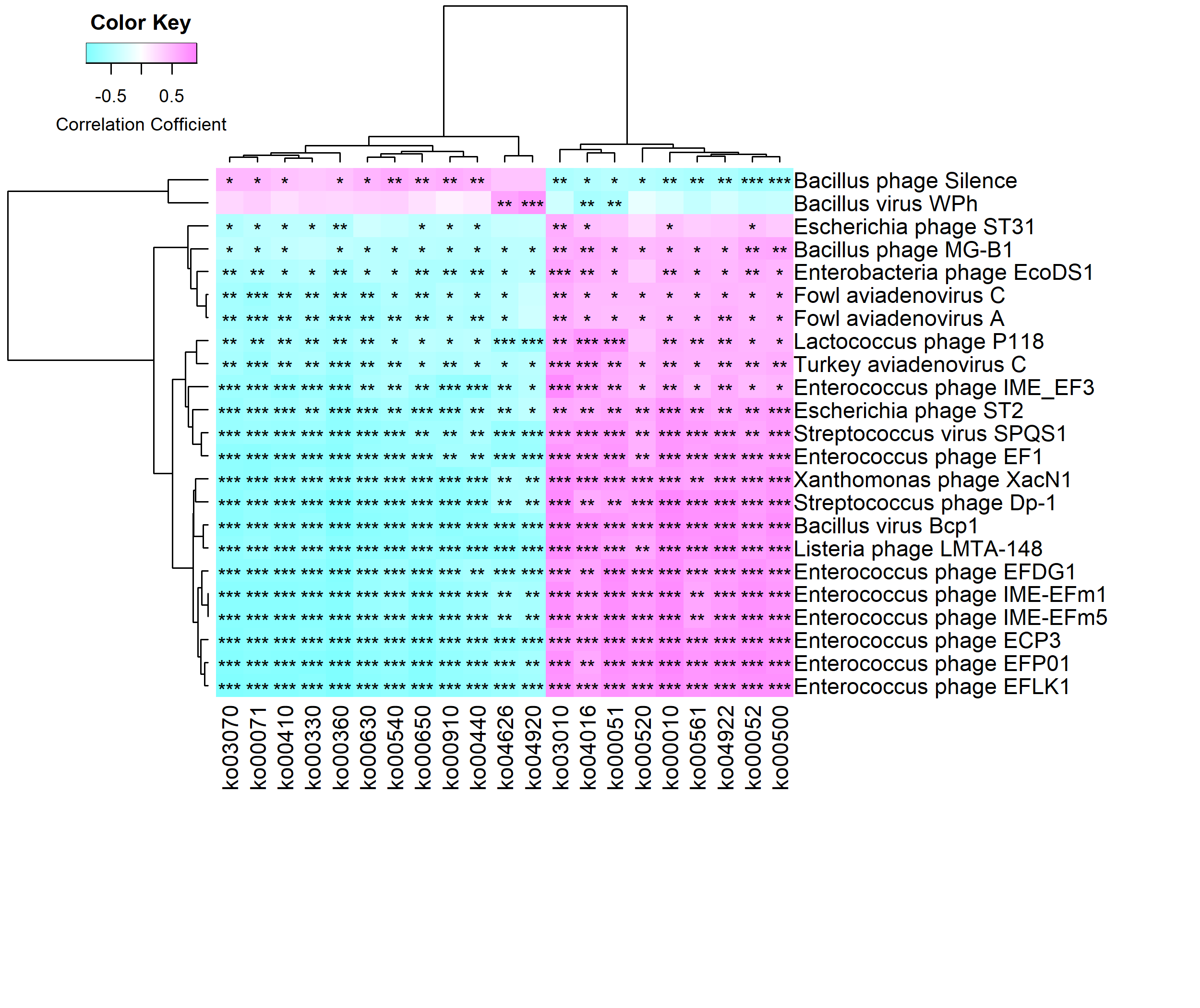


**Supplementary Figure 2** Heat maps showing the relationships between differential gut bacteriophage species and differential KEGG pathways. The X-axis represents the KEGG pathways. The Y-axis indicates the bacteriophage species. * *P* < 0.05, ** *P* < 0.01, and *** *P* < 0.001.


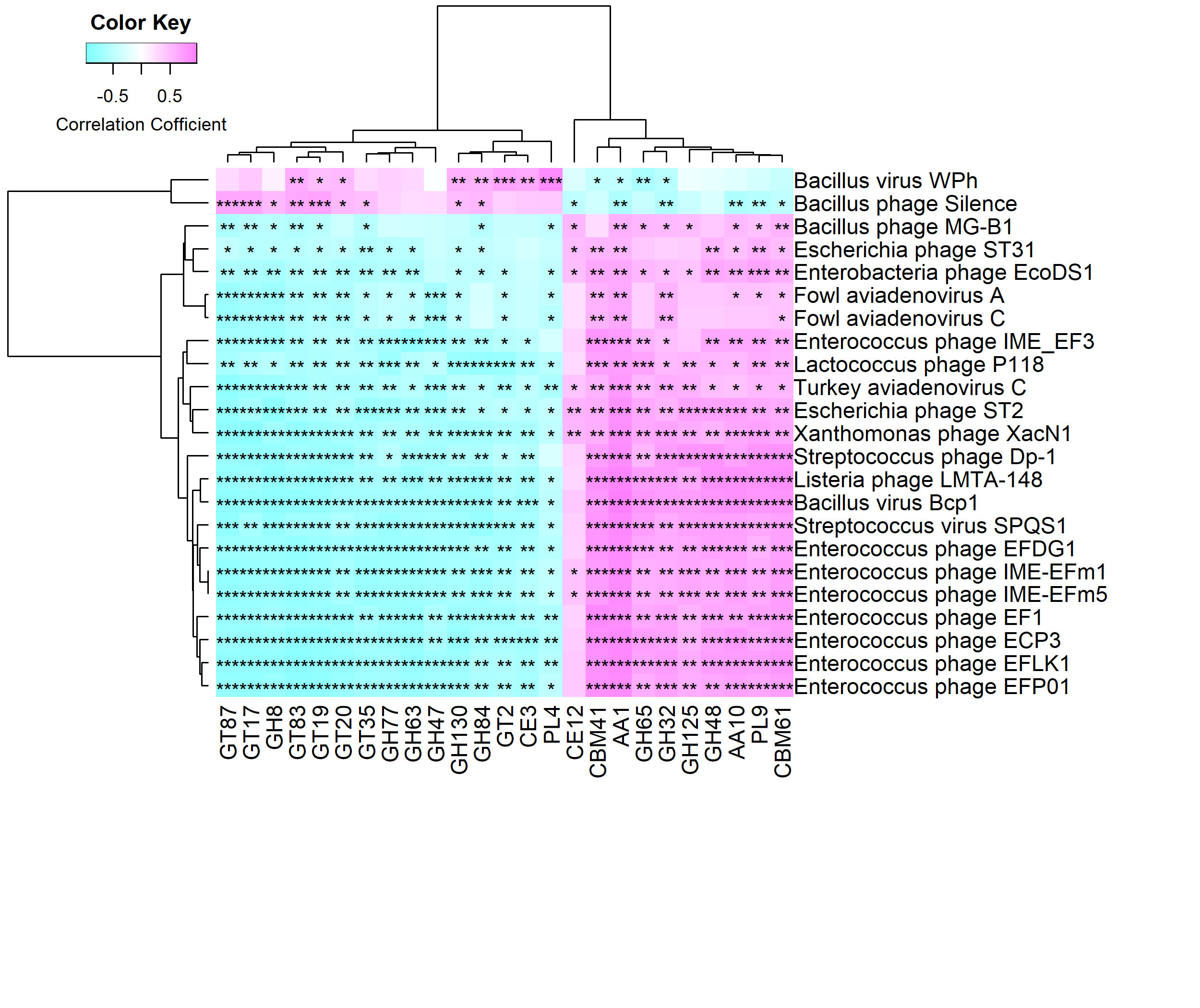


**Supplementary Figure 3** Heat maps showing the relationships between differential gut bacteriophage species and differential CAZymes. The X-axis represents the CAZymes. The Y-axis indicates the bacteriophage species. * *P* < 0.05, ** *P* < 0.01, and *** *P* < 0.001.

**Supplementary Table 1 Gut bacteriophages showing significantly different abundances between the day 20 and day 70 groups using LEfSe analysis.**

| **Species level** | **Group** | **LDA value** | ***P* value** |
| --- | --- | --- | --- |
| *Enterococcus phage EFP01* | day20 | 4.82 | 9.10E-04 |
| *Enterococcus phage IME-EFm5* | day20 | 4.64 | 1.12E-03 |
| *Enterococcus phage IME-EFm1* | day20 | 4.55 | 1.12E-03 |
| *Enterococcus phage EFDG1* | day20 | 4.51 | 2.78E-03 |
| *Enterococcus phage vB_EfaP_IME199* | day20 | 4.07 | 5.34E-03 |
| *Fowl aviadenovirus C* | day20 | 4.07 | 2.75E-03 |
| *Streptococcus phage P5652* | day20 | 4.04 | 7.65E-03 |
| *Rhodococcus virus Poco6* | day70 | 4.00 | 5.62E-03 |
| *Enterococcus phage EF1* | day20 | 3.84 | 9.78E-04 |
| *Escherichia phage vB_Eco_swan01* | day20 | 3.51 | 8.91E-03 |
| *Bacillus virus WPh* | day70 | 3.44 | 5.43E-03 |
| *Streptococcus phage Dp-1* | day20 | 3.41 | 5.25E-03 |
| *Marinobacter phage PS6* | day70 | 3.39 | 5.76E-03 |
| *Bacillus phage Silence* | day70 | 3.38 | 8.03E-04 |
| *Escherichia phage ECD7* | day20 | 3.36 | 5.76E-03 |
| *Lactococcus phage P118* | day20 | 3.33 | 3.78E-03 |
| *Enterococcus phage phiSHEF4* | day20 | 3.27 | 4.35E-03 |
| *Cronobacter phage S13* | day70 | 3.26 | 2.23E-03 |
| *Croceibacter phage P2559Y* | day70 | 3.25 | 4.63E-03 |
| *Enterococcus phage IME_EF3* | day20 | 3.23 | 3.09E-03 |
| *Enterococcus phage ECP3* | day20 | 3.20 | 1.24E-03 |
| *Vibrio phage vB_VorS-PVo5* | day20 | 3.18 | 2.23E-03 |
| *Enterococcus phage EFLK1* | day20 | 3.16 | 4.37E-04 |
| *Escherichia phage G AB-2017* | day20 | 3.15 | 8.03E-04 |
| *Enterobacteria phage 285P* | day20 | 3.15 | 2.23E-03 |
| *Lactobacillus phage Lrm1* | day20 | 3.15 | 2.23E-03 |
| *Enterococcus virus BC611* | day20 | 3.12 | 5.76E-03 |
| *Salmonella phage BPS11Q3* | day20 | 3.09 | 8.03E-04 |
| *Enterobacteria phage EcoDS1* | day20 | 3.03 | 8.03E-04 |
| *Streptococcus virus SPQS1* | day20 | 3.00 | 1.70E-03 |
| *Escherichia phage P AB-2017* | day20 | 2.99 | 5.76E-03 |
| *Bacillus phage MG-B1* | day20 | 2.98 | 6.43E-03 |
| *Cronobacter virus PBES02* | day20 | 2.97 | 2.23E-03 |
| *Enterococcus phage AUEF3* | day20 | 2.95 | 8.03E-04 |
| *Turkey aviadenovirus C* | day20 | 2.94 | 6.93E-05 |
| *Bacillus phage vB_BhaS-171* | day20 | 2.91 | 8.80E-03 |
| *Kluyvera virus Kvp1* | day20 | 2.88 | 5.76E-03 |
| *Shigella virus Shfl1* | day20 | 2.84 | 8.91E-03 |
| *Xanthomonas phage XacN1* | day20 | 2.83 | 7.34E-05 |
| *Fowl aviadenovirus A* | day20 | 2.79 | 3.43E-03 |
| *Bacillus virus Bcp1* | day20 | 2.79 | 2.66E-04 |
| *Escherichia phage vB_EcoP_F* | day20 | 2.79 | 5.76E-03 |
| *Enterococcus phage vB_EfaS_IME196* | day20 | 2.76 | 8.39E-03 |
| *Vibrio phage pVco-5* | day20 | 2.76 | 5.58E-03 |
| *Enterococcus phage VPE25* | day20 | 2.76 | 2.23E-03 |
| *Escherichia phage L AB-2017* | day20 | 2.73 | 3.61E-03 |
| *Vibrio phage phi 1* | day20 | 2.71 | 5.73E-03 |
| *Streptococcus phage phiJH1301-2* | day20 | 2.68 | 2.23E-03 |
| *Clostridium phage phiCT453A* | day20 | 2.67 | 2.23E-03 |
| *Listeria phage LMTA-148* | day20 | 2.67 | 8.03E-04 |
| *Enterococcus phage VFW* | day20 | 2.64 | 8.03E-04 |
| *Escherichia phage ST31* | day20 | 2.62 | 2.23E-03 |
| *Streptococcus phage phi-m46.1* | day20 | 2.60 | 5.76E-03 |
| *Staphylococcus virus G1* | day20 | 2.60 | 2.23E-03 |
| *Escherichia phage ST2* | day20 | 2.56 | 6.59E-04 |

**Supplementary Table 2 Gut bacteriophages showing significantly different abundances between the day 20 and day 70 groups through random forest analysis.**

| **Species level** | **MeanDecreaseAccuracy** | **MeanDecreaseGini** |
| --- | --- | --- |
| *Turkey aviadenovirus C* | 7.66 | 0.58 |
| *Escherichia phage vB_EcoS-MY* | 6.36 | 0.42 |
| *Enterococcus phage IME-EFm5* | 5.32 | 0.34 |
| *Xanthomonas phage XacN1* | 5.23 | 0.29 |
| *Enterococcus phage IME-EFm1* | 4.79 | 0.27 |
| *Bacillus virus BCP82* | 4.78 | 0.29 |
| *Fowl aviadenovirus C* | 4.50 | 0.18 |
| *Enterococcus phage EF1* | 4.41 | 0.24 |
| *Enterococcus phage EFP01* | 4.28 | 0.21 |
| *Fowl aviadenovirus A* | 4.25 | 0.16 |
| *Enterococcus phage EFDG1* | 4.08 | 0.19 |
| *Enterococcus phage EFLK1* | 3.77 | 0.20 |
| *Bacillus virus Bcp1* | 3.66 | 0.16 |
| *Bacillus phage MG-B1* | 3.55 | 0.16 |
| *Streptococcus phage Dp-1* | 3.36 | 0.18 |
| *Enterococcus phage ECP3* | 3.18 | 0.10 |
| *Pacmanvirus A23* | 3.14 | 0.13 |
| *Streptococcus virus SPQS1* | 3.09 | 0.16 |
| *Enterococcus phage IME_EF3* | 2.98 | 0.09 |
| *Streptococcus virus Sfi21* | 2.96 | 0.10 |
| *Enterobacteria phage EcoDS1* | 2.94 | 0.10 |
| *Lactococcus phage P118* | 2.87 | 0.12 |
| *Listeria phage LMTA-148* | 2.80 | 0.07 |
| *Bacillus phage Silence* | 2.77 | 0.11 |
| *Escherichia phage ST2* | 2.72 | 0.10 |
| *Bacillus virus NIT1* | 2.60 | 0.09 |
| *Bacillus virus WPh* | 2.56 | 0.07 |
| *Escherichia phage ST31* | 2.51 | 0.09 |

**Supplementary Table 3 The 23 overlapping gut bacteriophages between the day 20 and day 70 groups through LEfSe and random forest analysis.**

| **Species level** | **Group** | **MeanDecreaseAccuracy** | **MeanDecreaseGini** | **LDA value** | ***P* value** |
| --- | --- | --- | --- | --- | --- |
| *Turkey aviadenovirus C* | day20 | 7.66 | 0.58 | 2.94 | 6.93E-05 |
| *Enterococcus phage IME-EFm5* | day20 | 5.32 | 0.34 | 4.64 | 1.12E-03 |
| *Xanthomonas phage XacN1* | day20 | 5.23 | 0.29 | 2.83 | 7.34E-05 |
| *Enterococcus phage IME-EFm1* | day20 | 4.79 | 0.27 | 4.55 | 1.12E-03 |
| *Fowl aviadenovirus C* | day20 | 4.50 | 0.18 | 4.07 | 2.75E-03 |
| *Enterococcus phage EF1* | day20 | 4.41 | 0.24 | 3.84 | 9.78E-04 |
| *Enterococcus phage EFP01* | day20 | 4.28 | 0.21 | 4.82 | 9.10E-04 |
| *Fowl aviadenovirus A* | day20 | 4.25 | 0.16 | 2.79 | 3.43E-03 |
| *Enterococcus phage EFDG1* | day20 | 4.08 | 0.19 | 4.51 | 2.78E-03 |
| *Enterococcus phage EFLK1* | day20 | 3.77 | 0.20 | 3.16 | 4.37E-04 |
| *Bacillus virus Bcp1* | day20 | 3.66 | 0.16 | 2.79 | 2.66E-04 |
| *Bacillus phage MG-B1* | day20 | 3.55 | 0.16 | 2.98 | 6.43E-03 |
| *Streptococcus phage Dp-1* | day20 | 3.36 | 0.18 | 3.41 | 5.25E-03 |
| *Enterococcus phage ECP3* | day20 | 3.18 | 0.10 | 3.20 | 1.24E-03 |
| *Streptococcus virus SPQS1* | day20 | 3.09 | 0.16 | 3.00 | 1.70E-03 |
| *Enterococcus phage IME_EF3* | day20 | 2.98 | 0.09 | 3.23 | 3.09E-03 |
| *Enterobacteria phage EcoDS1* | day20 | 2.94 | 0.10 | 3.03 | 8.03E-04 |
| *Lactococcus phage P118* | day20 | 2.87 | 0.12 | 3.33 | 3.78E-03 |
| *Listeria phage LMTA-148* | day20 | 2.80 | 0.07 | 2.67 | 8.03E-04 |
| *Bacillus phage Silence* | day70 | 2.77 | 0.11 | 3.38 | 8.03E-04 |
| *Escherichia phage ST2* | day20 | 2.72 | 0.10 | 2.56 | 6.59E-04 |
| *Bacillus virus WPh* | day70 | 2.56 | 0.07 | 3.44 | 5.43E-03 |
| *Escherichia phage ST31* | day20 | 2.51 | 0.09 | 2.62 | 2.23E-03 |

**Supplementary Table 4 Ingredient composition of diet for all quails.**

| Ingredient, % | diet |
| --- | --- |
| Maize | 54 |
| Soybean meal | 15 |
| Wheat bran | 3.5 |
| Rice bran | 10 |
| Fish meal | 15 |
| Bone meal | 1.5 |
| Hay meal | 1 |
| Total | 100 |
| Composition |  |
| Digestible energy (MJ/kg) | 13.56 |
| Crude protein (%) | 22.76 |
| Calcium (%) | 0.91 |
| Phosphorus (%) | 0.63 |
| Lysine (%) | 1.25 |
| Methionine (%) | 0.43 |
